# Editorial: Idiopathic pulmonary fibrosis: epidemiology, prognosis and treatment

**DOI:** 10.3389/fmed.2023.1195263

**Published:** 2023-06-01

**Authors:** Antonella Caminati, Federica Meloni, Masaki Fujita

**Affiliations:** ^1^San Giuseppe Hospital, IRCCS MultiMedica, Milan, Italy; ^2^University of Pavia & Fondazione IRCCS Policlinico San Matteo, UOS Transplant Center, Pavia, Italy; ^3^Department of Respiratory Medicine, Fukuota University Hospital, Fukuoka, Japan

**Keywords:** IPF, epidemiology, therapy, gender, genetic predisposition

Idiopathic pulmonary fibrosis is a rare disease with a chronic progressive course that invariably leads to respiratory failure and death (1). Many aspects need to be clarified regarding the epidemiology, prognosis, and treatment of this complex disease. In addition, the pathogenesis of IPF remains partially obscure. In fact, while senescence has been shown to play a crucial role, and some genetic determinants have been described, we still need to clarify the exact molecular mechanisms underlying all the steps which lead to epithelial cell death and fibrogenesis, as well as the exact role of inflammation and immunity. Some of these issues have been explored through original papers published in this journal. Dai et al. used bioinformatics methods to analyze the differentially expressed genes in both healthy and IPF patients, and to elucidate the signaling pathways and key gene targets closely related to IPF development. The Authors found 43 distinctly expressed genes in IPF and suggested that CRTAC1, COL10A1, COMP, IGFL2, NECAB1, SCG5, and SLC6A4 E SPP1 (associated with monocytes, plasma cells, neutrophils, and regulatory T cells) may be used as diagnostic markers for IPF. Li et al. investigated the role of ferroptosis-related genes (FRGs) in bronchoalveolar lavage (BAL) fluid as prognostic biomarkers in IPF. Ferroptosis is a previously unrecognized non-apoptotic form of regulated cell death that is iron-dependent and characterized by increased lipid-peroxidation. Elevated iron levels have been described in IPF patients, along with airway fibrosis and altered pulmonary function. It has, therefore, been hypothesized that ferroptosis may be involved in IPF pathogenesis (2). FRGs are genes defined as markers or regulatory factors of ferroptosis. The Authors found 19 FRGs differentially expressed in BAL from IPF and healthy controls that were associated with prognosis (Li et al.). Within these, five genes were identified as a high risk signature. Patients with a high risk signature showed an overall survival rate that was significantly lower when compared to patients with a low risk profile.

The development of IPF is also linked to exposure to smoke and pollution as being external triggers for lung injury, inflammation, and abnormal repair mechanisms. Recently, dyslipidemia has been associated with the pathogenesis of inflammation, interstitial lung injury, and fibrosis (3), thus suggesting a protective role of statins in view of their use as anti-inflammatory and immunoregulatory drugs (4, 5).

The combined simvastatin and ezetimibe therapy demonstrated a significant reduction in the secretion of the pro-inflammatory cytokine IL-1b from monocytes in hypercholesterolemia patients (6). Seenak et al., in a study on an animal model, showed a protective effect of atorvastatin and ezetimibe treatment against hypercholesterolemia-induced pulmonary fibrosis in rats.

IPF is characterized by a male predominance. In different real-life data, males account for 60–70% of total cases (7, 8). In a recent study it has been found that clinicians rarely assign a diagnosis of IPF to women, and gender is the most discriminating pre-test diagnostic criteria (9). However, few studies have considered gender-related features and outcomes in IPF. Sesé et al. performed a multicenter national prospective cohort study with a 5-year follow-up to analyze gender differences in IPF. The authors found 236 patients with a new diagnosis of IPF between 2007 and 2010, 78% of whom were males (Sesé et al.). In this study, women were diagnosed at an early stage of the disease with a more preserved lung function, and radiologically showed less honeycombing and emphysema as compared to men. Women showed less frequent exposure to smoke and inhaled aerocontaminants, and were more likely to be over 65 years of age. Disease progression and overall survival remained comparable regardless of gender in this study, but this aspect is debated in the literature (8, 10). Women also have less access to lung transplantation. In recent years the introduction of antifibrotic drugs (pirfenidone and nintedanib) were shown to reduce disease progression in IPF patients and to stabilize the disease course. Real world survival data from large unselected and untreated IPF patients are scarce. Cottin et al., in a retrospective population-based cohort study, used the claims data from the French National Health System to describe the outcome of IPF patients with an IPF diagnosis who did not receive antifibrotic drugs in the period 2015–2016. A total of 5,360 patients (43.2% of all patients with a diagnosis of IPF) not treated with antifibrotics were included in this study and showed a 50% all-cause mortality and a 42% cumulative incidence rate of acute respiratory-related hospitalization at 3 years. This observation confirms the poor prognosis and outcome of IPF. Another relevant issue relates to the influence of a diagnostic path on IPF epidemiology. Di Bidino et al. performed a retrospective, population-based longitudinal 5-year study that involved all four IPF reference centers in the Lazio region (in Italy) to assess whether the hub-based model is effective in the early diagnosis and therapeutic management of the disease. An early, correct, and timely diagnosis and subsequent treatment of IPF are fundamental in order to improve outcomes in this complex disease. The Authors found that the number of new diagnoses of IPF significantly increased during the study period and that the number of patients eligible for antifibrotic treatment increased, suggesting that the patients were diagnosed at an early stage of the disease (Di Bidino et al.). The study demonstrated that this model of care significantly increases the ability to detect IPF cases, and to identify the key players of disease management based on the four qualified IPF centers. The protagonists of this care system are general practitioners, pulmonologists, and IPF centers supported by telemedicine. This represents the first case of a regional Diagnostic-Therapeutic Care Pathways involving different health professionals, including those external to the IPF centers, supported by digital solutions, and represents a possible model for future disease management.

Pan et al. investigated the prognostic impact of marital status on malignant pleural mesothelioma (MPM), a rare neoplastic disease, and found that marital status is an independent favorable prognostic factor. In this study of 3,997 patients with MPM, the Authors observed that married groups had a lower risk of death and, after adjustment for demographic and tumor features, had a 13% reduction of death compared with unmarried patients. This study outlined that social conditions are also important factors in the management of disease and its outcome.

## Author contributions

All authors listed have made a substantial, direct, and intellectual contribution to the work and approved it for publication.
